# Estradiol Regulation of Nucleotidases in Female Reproductive Tract Epithelial Cells and Fibroblasts

**DOI:** 10.1371/journal.pone.0069854

**Published:** 2013-07-25

**Authors:** Zheng Shen, John V. Fahey, Jack E. Bodwell, Marta Rodriguez-Garcia, Richard M. Rossoll, Sarah G. Crist, Mickey V. Patel, Charles R. Wira

**Affiliations:** Department of Physiology and Neurobiology, Geisel School of Medicine at Dartmouth, Lebanon, New Hampshire, United States of America; University of Toronto, Canada

## Abstract

The use of topical and oral adenosine derivatives in HIV prevention that need to be maintained in tissues and cells at effective levels to prevent transmission prompted us to ask whether estradiol could influence the regulation of catabolic nucleotidase enzymes in epithelial cells and fibroblasts from the upper and lower female reproductive tract (FRT) as these might affect cellular TFV-DP levels. Epithelial cells and fibroblasts were isolated from endometrium (EM), endocervix (CX) and ectocervix (ECX) tissues from hysterectomy patients, grown to confluence and treated with or without estradiol prior to RNA isolation. The expression of nucleotidase (NT) genes was measurable by RT-PCR in epithelial cells and fibroblasts from all FRT tissues. To determine if sex hormones have the potential to regulate NT, we evaluated NT gene expression and NT biological activity in FRT cells following hormone treatment. Estradiol increased expression of Cytosolic 5′-nucleotidase after 2 or 4 h in endometrial epithelial cells but not epithelial cells or fibroblasts from other sites. In studies using a modified 5′-Nucleotidase biological assay for nucleotidases, estradiol increased NT activity in epithelial cells and fibroblasts from the EM, CX and ECX at 24 and 48 h. In related studies, HUVEC primary cells and a HUVEC cell line were unresponsive to estradiol in terms of nucleotidase expression or biological activity. Our findings of an increase in nucleotidase expression and biological activity induced by estradiol do not directly assess changes in microbicide metabolism. However, they do suggest that when estradiol levels are elevated during the menstrual cycle, FRT epithelial cells and fibroblasts from the EM, CX and ECX have the potential to influence microbicide levels that could enhance protection of HIV-target cells (CD4+T cells, macrophages and dendritic cells) throughout the FRT.

## Introduction

Thirty years into the Human Immunodeficiency Virus (HIV) global pandemic, more than 30 million people have died with an additional 33 million presently living with HIV [1], [2]. Worldwide, approximately 70% of all new cases are spread by sexual intercourse, with women more likely to be infected than men [3]. Vaginal and anal sexual intercourse are the primary sources of infection in women, with adolescent age, sexual violence, and co-infection with sexually transmitted diseases (STDs) among the risk factors that contribute to enhanced susceptibility to HIV infection [2], [4].

With no effective vaccine available, attention has been focused on the use of anti-retroviral drugs to prevent infection (Pre-exposure Prophylaxis (PrEP)). For example, the nucleoside-analog reverse transcriptase inhibitor (NRTI) tenofovir demonstrated efficacy in in vitro studies, animal models and initial clinical trials [5], [6]. When delivered orally, tenofovir (TFV) accumulated in rectal tissue at a 33-fold higher concentration than in plasma, thus having the potential to inhibit the establishment of a founder population of infected cells at the site of HIV introduction during anal sex [5]. Topical application of microbicide gels to the GI and genital mucosa specific sites has also been effective in reducing infection. For example, the Centre for the AIDS Programme of Research in South Africa (CAPRISA 004, a phase IIb study), demonstrated a 39% efficacy of the Tenofovir gel used vaginally before and after sex in reducing the risk of HIV acquisition among women [7]. However, in direct contrast, the use of oral TFV and TFV as a vaginal gel in the Vaginal and Oral Interventions to Control the Epidemic (VOICE) trial [8] failed to protect women against the sexual acquisition of HIV. As a result, both oral and vaginal TFV arms of the VOICE trial were terminated [9] and subsequent analysis revealed a serious lack of adherence [10]. While adherence in trials is critical to evaluating success or failure, other factors such as hormonal status and existing STI may contribute as well.

The FRT is the primary mucosal site of infection by STDs including HIV. The FRT mucosa is composed of multiple cell types including epithelial cells, fibroblasts and immune cells. They all play a central role in providing cellular, humoral, and innate immune protection against bacterial and viral invasion [11], [12]. Previously, we found that FRT epithelial cells and fibroblasts were capable of both mounting an immune response and modulating immune cell function [13]–[18]. In addition, the secretion of immune factors by these FRT cells is under hormonal control [13]–[18]. Acting directly via hormone receptors and indirectly through cytokines, chemokines, and growth factors, estradiol and progesterone selectively enhance and suppress elements of the immune system during the menstrual cycle to optimize conditions for reproductive success [19]. By inhibiting immune responses to sperm and a non-syngeneic fetus during the secretory phase of the menstrual cycle, the chances for conceptus/fetus survival is increased. These changes led to the hypothesis of a “Window of Vulnerability” in the FRT, when HIV and other sexually transmitted pathogens are most likely to infect women [19]. Evidence (proof of concept) for the “Window” has been reported in a recent study with macaques. Using repeated, low dose SHIVSF162P3 vaginal exposures during normal menstrual cycles, 18 macaques (95%) first displayed viremia in the follicular phase compared with 1 macaque (5%) in the luteal phase. Taking into account a viral eclipse of 7–14 days before viremia could be detected, Vishwanathan et.al. estimated a “window” of most frequent viral transmission between days 24 and 32 of the menstrual cycle, when progesterone levels are high [20].

Tenofovir is an acyclic nucleotide analogue that is active only after entering target cells, where it is phosphorylated to TFV-diphosphate (TFV-DP) [21]. TFV-DP is a nucleotide reverse transcriptase inhibitor (NRTI) that causes chain termination of the growing DNA molecule. 5′-Nucleotidases are a class of enzymes involved in the catabolism of nucleotides through dephosphorylation of nucleotide terminal phosphate with a preference for nucleotide monophosphates [22]. These enzymes regulate the activation of the nucleotide analogs such as TFV, which need nucleotide kinases for phosphorylation to their active form. Several studies have demonstrated that nucleotidases are present in epithelial cells in the rodent FRT [23] and that nucleotidase mRNA levels are highest at estrus, the stage of the reproductive cycle when estrogens are elevated [24]. However, expression patterns and hormonal regulation of human nucleotidases in the FRT are unknown. Others have demonstrated that nucleotidases are present in vascular endothelial cells [25]. In human studies, breast cancer cell nucleotidase levels were higher in tumors lacking estrogen receptors (ER) than in tumors that were ER positive and known to be estradiol responsive [26].

The 5′-nucleotidases catalyze dephosphorylation of nucleotides with a preference for monophosphates and play a role in the regulation of nucleotide and nucleoside levels in all cells. These enzymes are of clinical interest due to their ability to inactivate of nucleoside analog drugs that are used in anticancer and antiviral drug therapy [27]–[29]. The 5′-nucleotidases vary in substrate specificity and tissue expression. For example, NT5E (CD73, 5′-ecto-nucleotidase), the most studied of this family of 5′-nucleotidases, catalyzes the conversion of purine 5′-mononucleotides such as AMP to nucleosides, is bound to the external surface of the plasma membrane in most tissues and has broad effects in cellular function, including cell attachment and transport of molecules into the cell (for review, see [22]). In addition, other forms of 5′-nucleotidases exist in the cytoplasm (C, such as NT5C1A) and mitochondria (M, such as NT5M).

Previous studies have demonstrated that the Organic Anion Transporter (OAT) proteins OAT1 and 3 are capable of transporting tenofovir into the cells lining the kidney proximal tubule and that specific members of the Multi-Drug Resistance Protein (MRP) transporter family can move tenofovir out of the cell and into the extracellular space [30], [31]. Others have shown that endothelial cells express OAT1 and OAT3 receptors and that androgen receptors in the brain are involved in the functional regulation of OAT3 at the blood brain barrier [32]. OAT1 and OAT3 receptors, known to transport TFV, are present in the rodent uterus [33] and thought to be under estrogen control based on gender differences in the kidney proximal tubule [34], [35]. Kohler and colleagues found that OAT1 and MRP4 have a direct role in transport and efflux of TFV, regulating levels in proximal tubules of the kidney [36].

Based on these findings, we hypothesized that estradiol has the potential to affect tenofovir efficacy by influencing NT expression and biological activity of epithelial cells, fibroblasts and endothelial cells in the human FRT. Our rationale for these studies is based on the recognition that to reach HIV-target cells imbedded in the stroma of the FRT, TFV taken orally must permeate endothelial cells to enter the FRT stroma, or in the case of vaginal deposition, move through and/or between epithelial cells, and fibroblasts to reach HIV-target cells (CD4+Tcells, macrophages and dendritic cells) in the upper and lower FRT [6]. The goal of this study was to determine whether estradiol and progesterone regulate the expression of transport receptors and 5′-nucleotidase enzyme expression and biological activity in epithelial cells and stromal fibroblasts throughout the FRT.

## Materials and Methods

### Source of Tissue

Human female reproductive tract tissues were obtained immediately following surgery from women who had undergone hysterectomies at Dartmouth-Hitchcock Medical Center (Lebanon, NH). All tissues used in this study, including Fallopian tube (FT), endometrium (EM), endocervical (CX), and ectocervical (ECX) tissues, were collected from patients with benign conditions such as fibroids and prolapse. Tissue samples were distal from the sites of pathology and were without pathological lesions as determined by a pathologist. All human subject work was carried out with the approval of the Dartmouth College Institutional review Board. Approval to use tissues was previously obtained from the Committee for the Protection of Human Subjects (CPHS), and with written informed consent obtained from the patient before surgery.

### Isolation of FRT Epithelial Cells and Fibroblasts

Epithelial cells and stromal fibroblasts from the FT, EM, CX and ECX were isolated as previously described [14], [37]. Briefly, tissues were rinsed with 1× HBSS with phenol red, containing 100 U/ml penicillin, 100 µg/ml streptomycin (all Thermo Scientific Hyclone, Logan, UT), and 0.35 mg/ml NaCO_3_ (Fisher Scientific, Pittsburgh, PA), then minced under sterile conditions into 1–2 mm fragments and digested at 37°C for 1 h using an enzyme mixture containing (final concentrations): 3.4 mg/ml pancreatin, 0.1 mg/ml hyaluronidase (both from Sigma, St. Louis, MO), 1.6 mg/ml collagenase D (Roche, Indianapolis, IN), and 2 mg/ml D-glucose (EMD, Gibbstown, NJ) in 1×HBSS (Invitrogen Life Technologies). Enzymes were chosen to maximize digestion of the extracellular matrix, as verified by microscopy of hematoxylin and eosin-stained frozen sections after digestion. After digestion, cells were dispersed through a 250-µm nylon mesh screen (Small Parts, Miami Lakes, FL), washed, and resuspended in complete media consisting of DMEM/F12 medium without phenol red, supplemented with 10 µM HEPES (both GIBCO, Life Technologies, Grand Island, NY), 100 µg/ml primocin (InvivoGen, San Diego, CA), 2 mM L-glutamine, 2.5% heat-inactivated defined fetal Bovine Serum (FBS) (both from ThermoScientific, Logan, UT) and 2.5% NuSerum (BD Biosciences, Bedford, MA). Epithelial cell sheets were separated from fibroblasts by filtration through a 20-µm mesh filter (Small Parts). Epithelial cell sheets were retained on the filter, while fibroblasts passed through. Epithelial cell sheets were recovered by rinsing and backwashing the filter with complete medium, centrifuged at 500×g for 5 min and analyzed for cell number and viability. Fibroblasts were centrifuged at 500×g for 10 min and resuspended in complete medium and placed in culture as described below.

### Isolation of Vaginal Epithelial Cells

Healthy, premenopausal women (n = 7) were recruited at Dartmouth-Hitchcock Medical Center (DHMC), Lebanon, New Hampshire. All volunteers were free of sexually transmitted infections and were not on any form of chemical or oral birth control or using an intra-uterine device. Volunteers were provided with an InStead© softcup (menstrual cup) to recover their vaginal fluid and epithelial cells. The menstrual cup was inserted into the vagina as per the manufacturer’s instructions for 1 h (http://www.softcup.com/video-tutorials). Upon removal at DHMC, the cup was placed in a 50 ml tube and immediately brought to the laboratory where its contents were centrifuged (800×g for 5 min) to separate cells and fluid. The cells were cultured in complete media and assayed for nucleotidases as described below.

### Peripheral Blood Mononuclear Cells (PBMC) and CD4+T Cells

PBMC were isolated by standard Ficoll density gradient centrifugation. CD4+T cells were purified from PBMC using magnetic negative selection with MACS kits (Miltenyi Biotech, Auburn, CA) and incubated in 6-well plates (Corning, Corning, NY) with X-VIVO 15 Media (Lonza, Walkersville,MD) supplemented with 10% human AB serum (Valley Biomedical, Winchester, VA). Purity higher than 98% was obtained for CD4+T cell populations after magnetic isolation (data not shown).

### Cell Incubation

#### Epithelial cell culture

To establish a cell culture system of polarized human FRT epithelial cells with both apical and basolateral compartments, FRT epithelial cells were cultured in Matrigel matrix (BD Biosciences) coated Falcon cell culture inserts in 24-well companion culture plates (Fisher Scientific). Apical and basolateral compartments contained 300 and 500 µl of complete medium respectively, which was changed every 2 days. Tight junction formation of cultured epithelial cell monolayers from FT, EM and CX was assessed by periodically measuring transepithelial resistance (TER) using an EVOM electrode and Voltohmmeter (World Precision Instruments, Sarasota, FL), as described previously [38]–[40].

#### Fibroblast cell culture

Fibroblasts were cultured in T75 flasks (Falcon, Fisher Scientific, Pittsburgh, PA) in complete medium. The medium was changed every 2 days for 4–6 days to remove non-adherent cells. Purity was verified by positive intracellular staining of vimentin and surface expression of CD90 and lack of CD45 [41], [42]. Once stromal fibroblasts reached confluence, they were trypsinized with 0.05% trypsin-EDTA (GIBCO, Life Technologies) and seeded into a 24-well plate at a density of 2×10^5^ cells per well in 500 µl complete medium for at least 48 h prior to treatment.

#### Vaginal epithelial cell culture

Freshly isolated vaginal epithelial cells were plated at 1×10^5^ cells per well in a 96-well culture plate (Fisher Scientific, Pittsburgh, PA) in 0.3 mL of complete media for 24 h prior to treatment.

#### Endothelial cell culture

Endothelial cells were obtained from American Type Culture Collection (ATCC; Manassas, VA). The CRL-1730 human endothelial cell line was cultured in flasks using F-12K medium supplemented with heparin, endothelial cell growth supplement (ATCC) and 10% fetal bovine serum. Primary human umbilical vein endothelial cells (HUVEC) were cultured in the same base media but supplemented with Endothelial Cell Growth Kit containing several growth factors (ATCC). Once endothelial cells reached confluence, they were trypsinized with 0.05% trypsin-EDTA (GIBCO, Life Technologies) and seeded into a 24-well plate at a density of 2×10^5^ cells per well in 500 µl complete medium for at least 48 h prior to treatment.

### Hormone Preparation

17β-estradiol (Calbiochem, Gibbstown, NJ) and progesterone (Calbiochem) was dissolved in 100% ethanol for an initial concentration of 1×10^−3^ M, evaporated to dryness and suspended in media containing 10% charcoal dextran-stripped FBS to a concentration of 1×10^−5^ M. Further dilutions were made to achieve final working concentrations of estradiol ranging from 1×10^−11^ to 1×10^−7^ M. Unless otherwise indicated, cells were treated with 5×10^−8^ M estradiol and/or 1×10^−7^ M progesterone. Both are standard hormone treatment concentrations used by our laboratory and each is within the physiological range of hormone concentration [43]. As a control, an equivalent amount of ethanol without dissolved hormone was initially evaporated. Cells in culture were switched to media containing charcoal dextran-stripped FBS prior to hormone treatment. After 24 h, the media was replaced and cells were treated with hormone. In all cases, hormone or ethanol control was added to both the apical and basolateral compartments for epithelial cell cultures and to plates for all other cells.

### TaqMan Real-time RT-PCR

Real-time PT-PCR was done with a two-step protocol as described previously [44]. Total RNA was isolated from cells using RNeasy reagent (Qiagen, Valencia, CA) and QIAshredder columns according to the manufacturer's recommendations (Qiagen), and purified on RNeasy columns (Qiagen) with on-column DNase digestion using the RNase-Free DNase set (Qiagen). For each specimen, 400 ng of total RNA was reverse-transcribed using the iScript cDNA synthesis kit (Bio-Rad) according to the manufacturer's recommendations. Relative mRNA expression levels of genes of interest were measured using the 5′ fluorogenic nuclease assay in real-time quantitative PCR using TaqMan chemistry on the ABI 7300 Prism real-time PCR instrument (Applied Biosystems, Carlsbad, CA). The 7 5′-Nucleotidases (ID nos. Hs01573922_m1, Hs00261369_m1, Hs00403674_m1, Hs00366992_m1, Hs00369454_m1, Hs01105359_g1, Hs00220234_m1,), β-actin (4333762F), OAT transporters 1 and 3 (Hs00537914_m1 and Hs00188599_m1), and progesterone receptor (Hs01556702) primer/MGB probe sets were obtained from Applied Biosystems assays-on-demand. PCR was conducted using the following cycle parameters: 95°C, 12 min for 1 cycle (95°C, 20 s; 60°C, 1 min), for 40 cycles. Analysis was conducted using the sequence detection software supplied with the ABI 7300. The software calculates the threshold cycle (C_t_) for each reaction and this was used to quantify the amount of starting template in the reaction. The C_t_ values for each set of duplicate reactions were averaged for all subsequent calculations. A difference in C_t_ values (ΔC_t_) was calculated for each gene by taking the mean C_t_ of each gene of interest and subtracting the mean C_t_ for the housekeeping gene β-actin for each cDNA sample. Assuming that each reaction functions at 100% PCR efficiency, a difference of one C_t_ represents a 2-fold difference. Relative expression levels were expressed as a fold-increase in mRNA expression and calculated using the formula 2^−ΔΔCt^.

### Measurement of 5′-Nucleotidase Biological Activity Assay

5′-nucleotidase biological activity was determined using a 5′-Nucleotidase kit (Diazyme Laboratories, Poway, CA) that was adapted from a serum to a cellular based assay by modifying the manufacturer’s protocol. Briefly, estradiol treated- or control-cells in culture were washed with Hepes Buffered Saline (0.15 M NaCl, 20 mM HEPES, pH7.4). Cells were dislodged with cell stripper (Cellgro, Manassas, VA), and permalized by incubating cells on ice 5–10 min in Hepes Buffered Saline containing 7.5 mM CHAPS (Research Organics, Cleveland, OH), followed by gentle pipetting. After adding reagent buffer to aliquots of permeabilized cells, the absorbance was monitored at 550 nm (25°C) on a SpectraMax, M5 (Molecular Devices Corporation, Sunnyvale, CA). Changes in absorbance were determined at 1 min intervals for ∼15 min and results over a seven-minute interval were averaged once the baseline had stabilized. A molar extinction coefficient of 18,440 M^−1 ^cm^−1^ was used to calculate enzyme activity (1 unit is equal to the production of 1 µmole of quinone dye/min) and standards were run with each assay to verify assay linearity. Sensitivity of this assay is 0.05 mU per million cells.

### Statistics

We have evaluated the data using column statistics – one sample t test, which compares the mean of every column of data to the hypothetical value (controls are by definition all equal to 1) using GraphPad Prism 5 (GraphPad Software, San Diego, CA). A p-value of <0.05 was taken as indicative of statistical significance.

## Results

### Human Epithelial Cells from the Upper and Lower FRT Express 5′-Nucleotidase Genes

**Figure 1:**
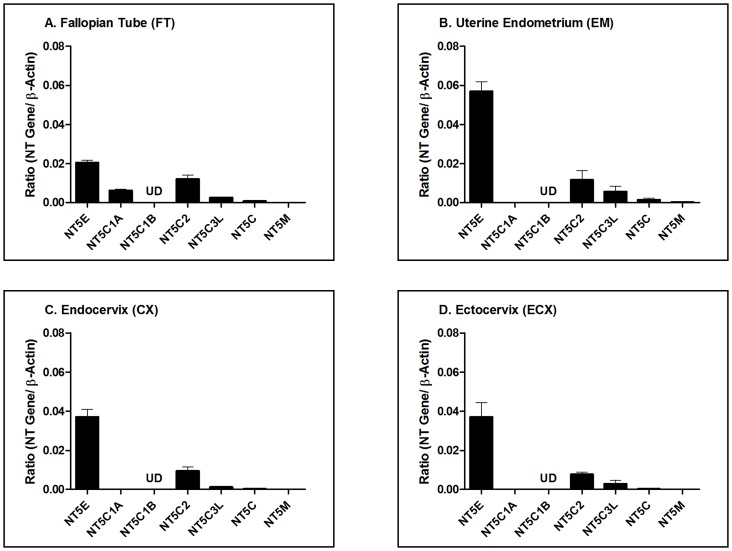
Relative gene expression of nucleotidases in FRT epithelial cells. Data is shown as the ratio of the nucleotidase (NT) gene expression to the expression of β-actin. Purified cultures of (A) FT n = 3, (B) EM n = 8, (C) CX n = 8 and (D) ECX n = 10 epithelial cells were analyzed for changes in nucleotidase gene expression by RT-PCR. n refers to the number of patients for each tissue. 5′-Nucleotidases measured were Ecto-5′-nucleotidase (NT5E), Cytosolic 5′-nucleotidase 1A (NT5C1A), Cytosolic 5′-nucleotidase 1B (NT5C1B), Cytosolic 5′-nucleotidase II (NT5C2), Cytosolic 5′-nucleotidase III (NT5C3L), Cytosolic 5′(3′)-deoxyribonucleotidase (NT5C), and Mitochondrial 5′(3′)-deoxyribonucleotidase (NT5M). UD = undetectable. The mean and SEM are shown.

Epithelial cells from the upper and lower FRT were analyzed for the gene expression of 5′-Nucleotidases by RT-PCR. 5′-Nucleotidases measured were Ecto-5′-nucleotidase (NT5E), Cytosolic 5′-nucleotidase 1A (NT5C1A), Cytosolic 5′-nucleotidase 1B (NT5C1B), Cytosolic 5′-nucleotidase II (NT5C2), Cytosolic 5′-nucleotidase III (NT5C3L), Cytosolic 5′(3′)-deoxyribonucleotidase (NT5C), and Mitochondrial 5′(3′)-deoxyribonucleotidase (NT5M). Total RNA was isolated from FRT cells and mRNA expression of 5′-Nucleotidases was examined by RT-PCR for quantitative comparison of the relative 5′-Nucleotidase expression. As shown in **Figure 1**, six out of seven nucleotidase genes analyzed were found in epithelial cells from the Fallopian tube (FT), Uterine endometrium (EM), Endocervix (CX), and Ectocervix (ECX). Of those nucleotidases analyzed, only NT5C1B was undetectable in all samples. When relative expression was analyzed, NT5E was expressed at the highest concentration in each cell type with endometrial epithelial cells having the greatest expression.

### Effect of Estradiol on Nucleotidase Gene Expression in FRT Epithelial Cells

**Figure 2:**
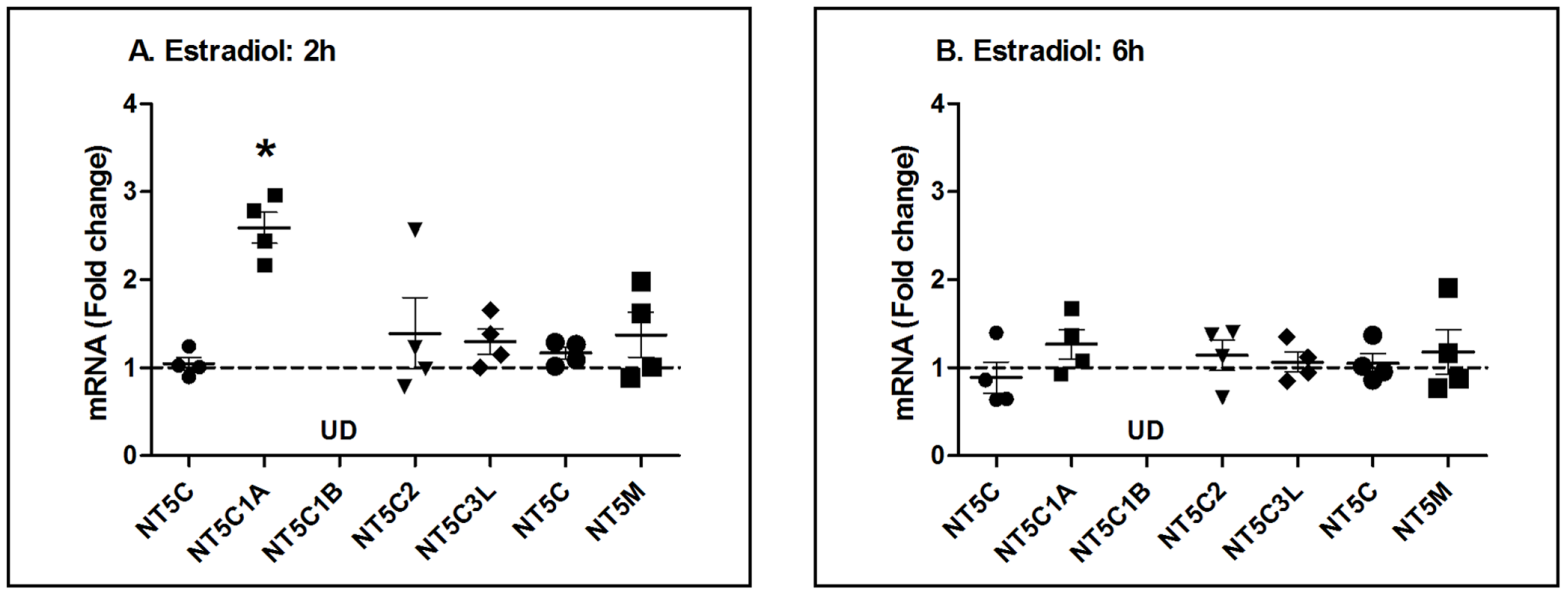
Effect of estradiol on nucleotidase gene expression in primary uterine endometrial epithelial cells. Fold change in mRNA expression compared to Control, which was assigned a value of 1 (dashed line), was analyzed by RT-PCR from purified cultures of polarized EM epithelial cells isolated from 4 patients and treated with estradiol (5×10^−8^ M) for (A) 2 h or (B) 6 h. NT enzymes are abbreviated as shown in legend to Figure 1. UD = undetectable. Mean and SEM are shown. *, P<0.05 represents significant difference between control and estradiol treatment for NT5C1A at 2 h.

**Figure 3:**
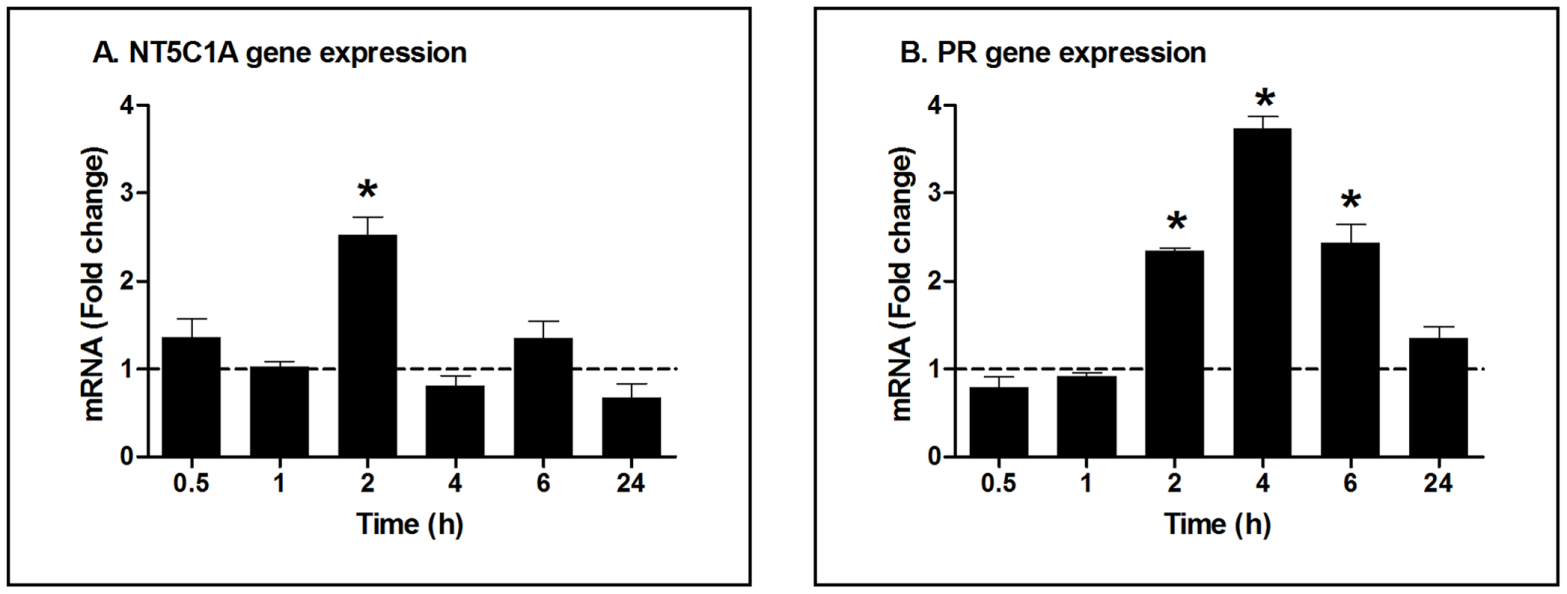
Detailed time course for effect of estradiol on NT5C1A and progesterone receptor gene expression in primary uterine endometrial epithelial cells. Fold change in (A) NT5C1A and (B) progesterone receptor mRNA expression analyzed by RT-PCR compared to control, which was assigned a value of 1 (dashed line), from purified cultures of polarized EM epithelial cells (patient number 6167) treated with estradiol (5×10^−8^ M) over time (30 mins, 1, 2, 4, 6 and 24 h). Mean and SEM are shown from 3 wells at each time point. *, P<0.05 represents significant difference between control and estradiol treatment.

To determine whether estradiol has an effect on nucleotidase gene expression in primary FRT epithelial cells, polarized cells were incubated with estradiol (5×10^−8^ M) for 2, 6 and 24 h. As seen in **Figure 2**, estradiol treatment increased the expression of NT5C1A at 2 h (**Figure 2A**) but not at 6 h (**Figure 2B**) in polarized epithelial cells from 4 patients with no measurable effect at 24 h (not shown) treatment. Interestingly, of the 7 genes analyzed, only NT5C1A responded to estradiol treatment with increased expression. To more fully define the pattern of nucleotidase expression, a detailed time course study was carried out in which polarized uterine epithelial cells, from a single patient (6167EM), were incubated with estradiol for ½ to 24 h. As seen in **Figure 3A**, in a detailed time course study, NT5C1A expression increased 2.5 fold at 2 h, after which it declined to control values at 4, 6 and 24 h. Since an increase in progesterone receptor (PR) expression serves as a positive control for estradiol responsiveness [45], [46], we evaluated PR expression in each of the cell preparations and found that PR expression increases by more than 2 fold at 2, 4 and 6 h (**Figure 3B**).

**Figure 4:**
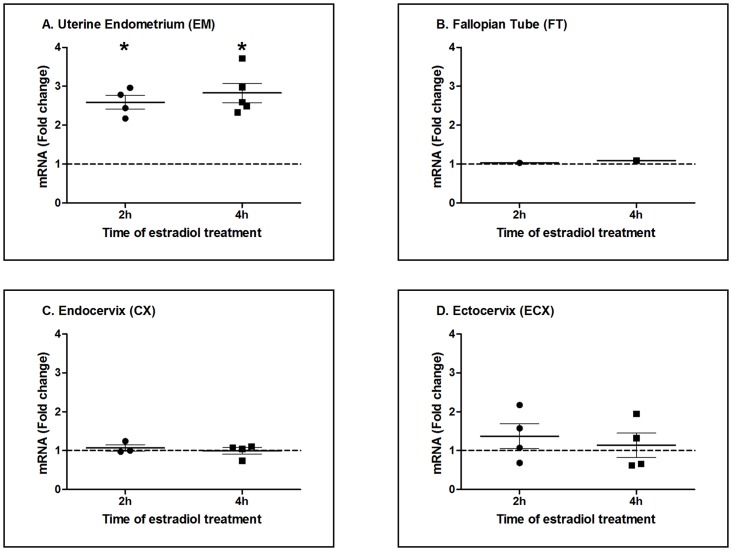
Estradiol effect on nucleotidase NT5C1A gene expression in FRT epithelial cells. Fold change in NT5C1A mRNA expression in epithelial cells from the (A) EM, n = 9 (B) FT, n = 2 (C) CX, n = 7 and (D) ECX, n = 8, treated with estradiol (5×10^−8^ M) for 2 (circles) or 4 h (squares). Control (no estradiol) is assigned a value of 1 (dashed line). n refers to the number of patients for each tissue. Mean and SEM are shown. *, P<0.05 represents significant difference between control and estradiol treatment for NT5C1A.

To determine the extent to which estradiol regulates nucleotidase expression in epithelial cells from the upper and lower FRT, polarized cells from the EM, FT, CX and ECX were incubated with E_2_ for either 2 or 4 h prior to analysis of all 7 nucleotidase genes. As shown in **Figure 4A**, in 9 patients, estradiol significantly increased NT5C1A expression in EM epithelial cells at 2 h (n = 4) or 4 h (n = 5), with only 1 cell preparation showing stimulation at both 2 and 4 h. In contrast to NT5C1A in the EM, we unexpectedly found that under identical incubation conditions, estradiol had no effect on gene expression of NT5C1A in epithelial cells from the FT (**Figure 4B**), CX (**Figure 4C**) or ECX (**Figure 4D**) at either 2 or 4 h. Further analysis of epithelial cells from FT, CX and ECX in time course studies (1–24 h) failed to show any evidence of an estradiol effect on nucleotidase gene expression on any of the 7 genes analyzed (data not shown).

**Figure 5:**
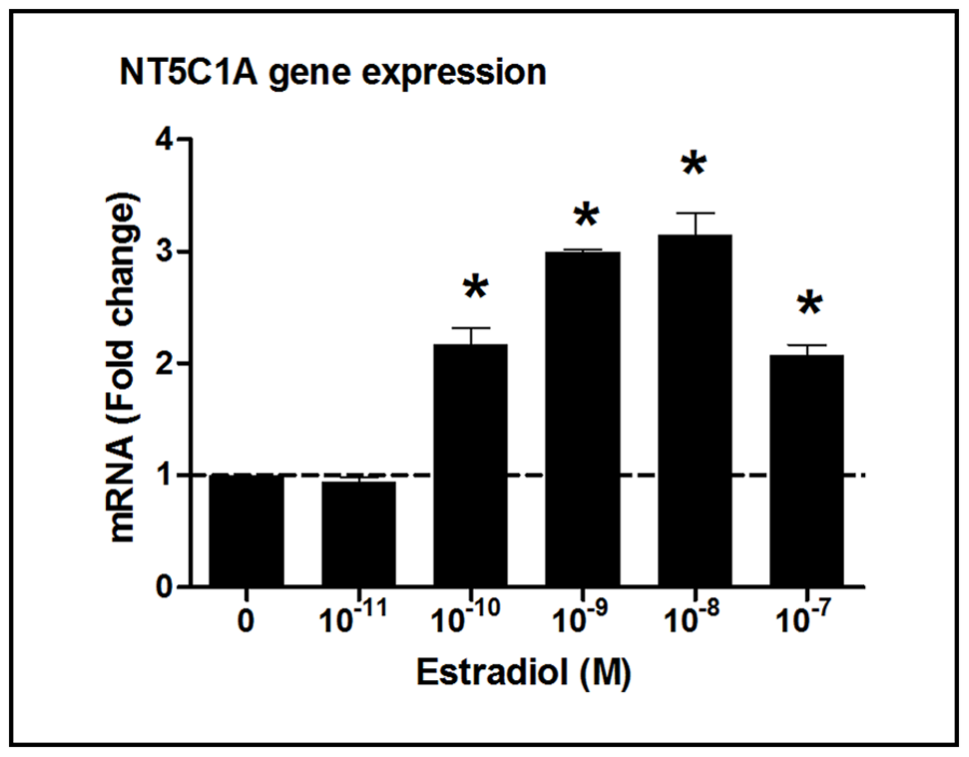
Estradiol has a dose-dependent effect on NT5C1A gene expression in primary uterine endometrial epithelial cells. Fold change in NT5C1A mRNA expression analyzed by RT-PCR from purified cultures of polarized EM epithelial cells (patient number 6157) treated with increasing doses of estradiol (from 1×10^−11 ^M up to 1×10^−7^ M) for 2 h. Control (no estradiol) is assigned a value of 1 (dashed line). The mean and SEM from triplicate cultures are shown. *, P<0.05 represents significant difference between control and estradiol treatment.

Given that estradiol levels in blood vary with stage of the menstrual cycle [43], we carried out a dose response experiment with EM epithelial cells from a single tissue (6157EM) at concentrations ranging from 1×10^−11^ M to 1×10^−7^ M for 2 h. As seen in **Figure 5**, estradiol had a stimulatory effect on NT5C1A gene expression (greater than 2-fold increase) in primary endometrium epithelial cells at doses ranging from 1×10^−10^ M to 1×10^−7^ M. The higher concentrations are the same concentrations at which the estrogen receptor is saturated [47].

### Nucleotidase Gene Expression in FRT Fibroblasts

**Figure 6:**
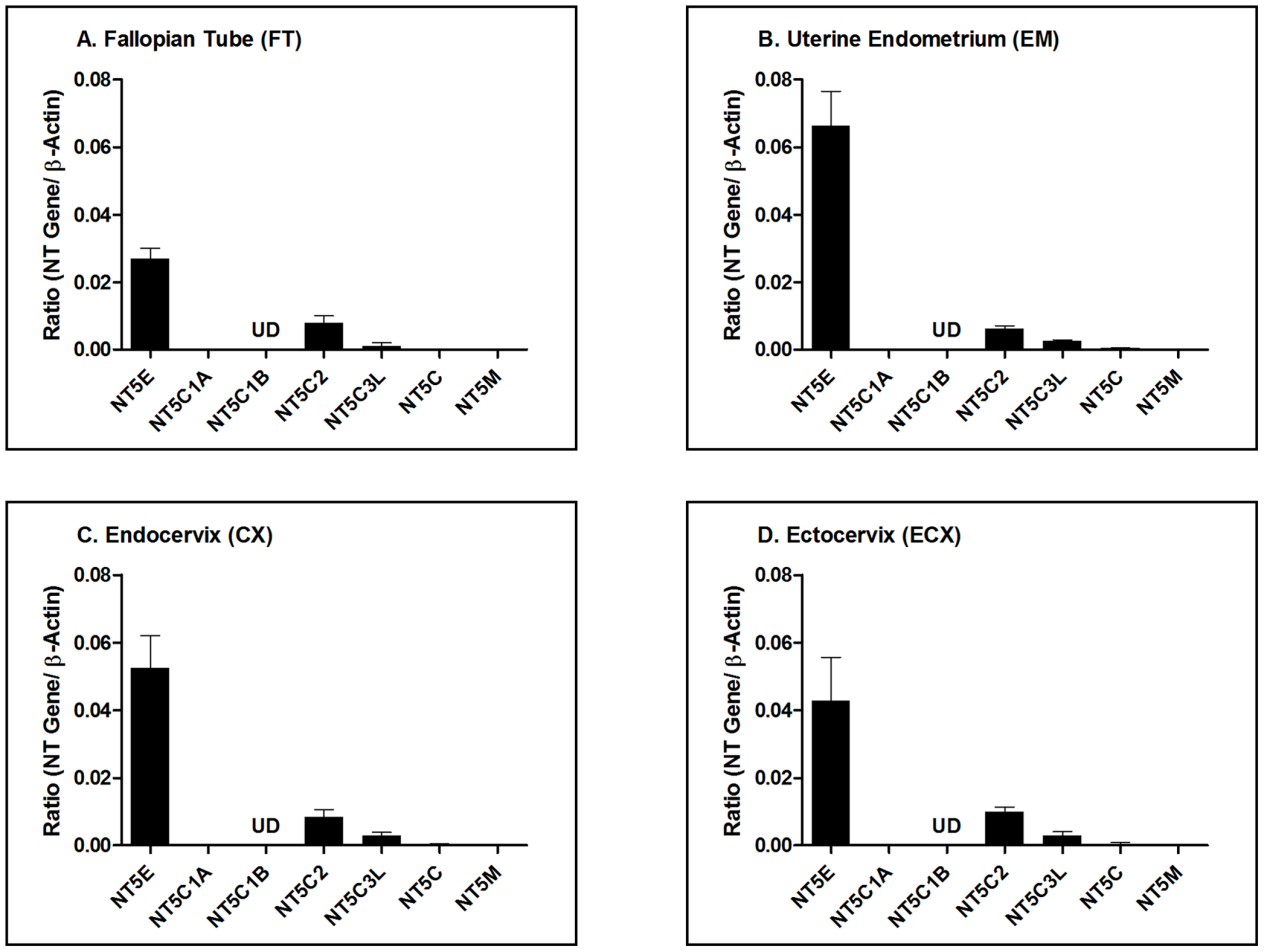
Relative gene expression of nucleotidases in FRT fibroblasts. Data is shown as the ratio of the nucleotidase (NT) gene expression to the expression of β-actin. Purified cultures of fibroblasts from (A) FT n = 2, (B) EM n = 7, (C) CX n = 6 and (D) ECX n = 5 were analyzed for changes in nucleotidase gene expression by RT-PCR. n refers to the number of patients. UD = undetectable. The mean and SEM are shown.

To determine whether underlying stromal fibroblasts from the upper and lower tract also express 5′-Nucleotidases, fibroblasts were isolated from FRT tissues and grown to confluence prior to analysis. Verification of cell purity was confirmed by flow cytometric staining (data not shown). Following the same procedure described above for RNA isolation and analysis of 5′-Nucleotidase genes, we found a profile of expression similar to that seen with epithelial cells. As seen in **Figure 6**, fibroblast expression of NT5E was significantly higher than any other gene analyzed. Interestingly, similar to that seen with epithelial cells (**Figure 1**), the second highest level of expression was NT5C2, with NT5C1B not detected in any of the 2 to 7 specimens analyzed. To evaluate whether estradiol alters nucleotidase mRNA expression, purified fibroblasts from the Fallopian tubes, uterus, endocervix and ectocervix were incubated with estradiol (5×10^−8^ M) for 2, 4, 6 and 24 h after which gene expression was analyzed. Irrespective of the time in culture, we found in 6–8 fibroblasts preparations, each from different patients, that estradiol had no significant effect on any of the genes expressed at any of the four sites (data not shown).

### Nucleotidase and OAT Transporter Gene Expression in Vaginal Epithelial Cells, Blood CD4+T cells and Endothelial Cells

#### Vaginal epithelial cells

Owing to the development of a protocol for isolating mRNA from vaginal epithelial cells, we have analyzed these cells for nucleotidase gene expression. Surprisingly, of the nucleotidase genes tested, only NT5C2 is expressed in squamous epithelial cells (data not shown). This is in contrast to epithelial cells from the FT, EM, CX and ECX, which expressed 6/7 of the genes analyzed.

#### CD4+T cells

**Figure 7:**
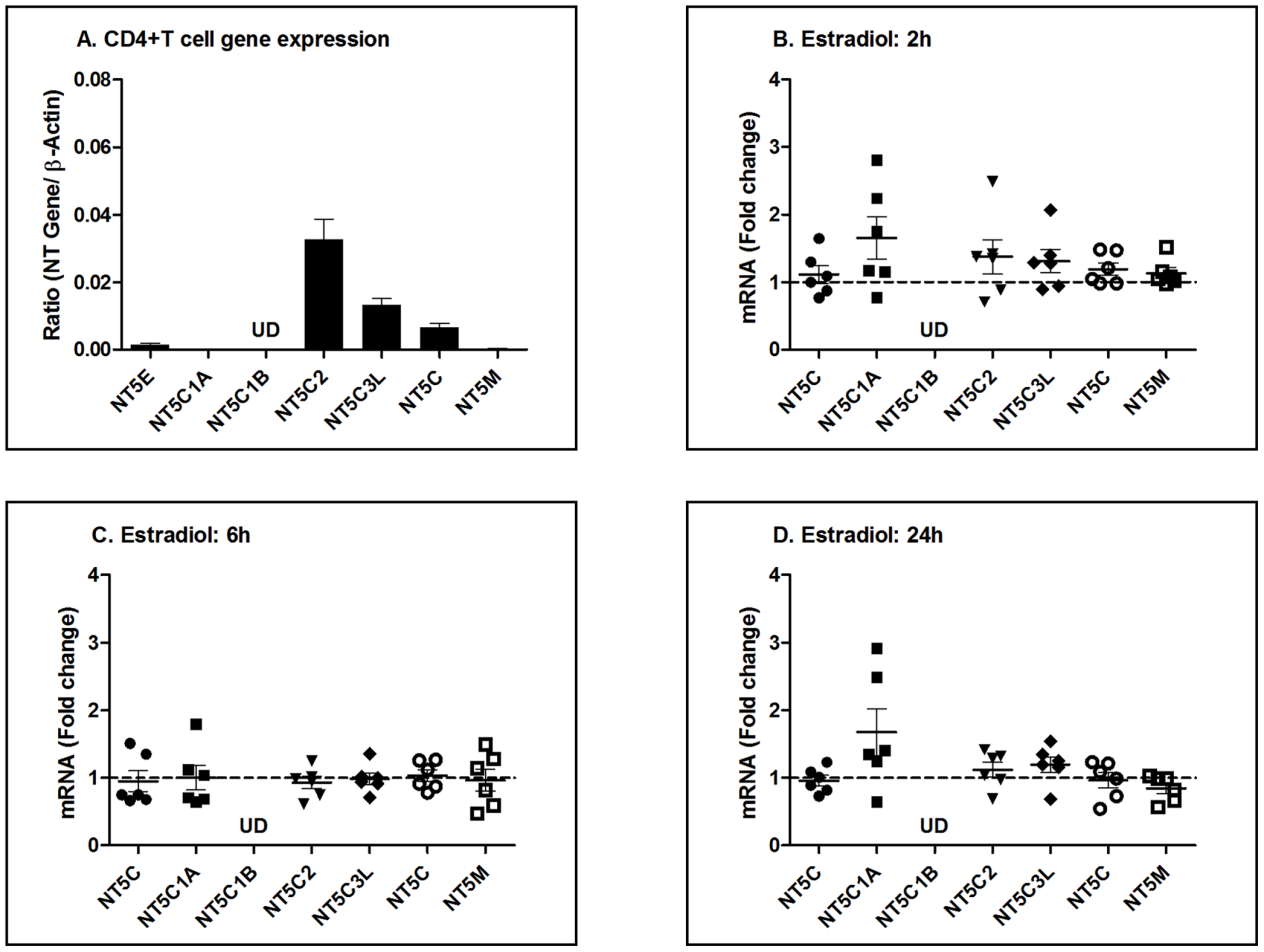
Effect of estradiol on nucleotidase gene expression in resting blood-derived CD4+T cells. (A) Relative gene expression of nucleotidases in blood-derived CD4+T Cells derived from 6 donors. Data is shown as the ratio of the nucleotidase (NT) gene expression to the expression of β-actin. (B–D) Effect of estradiol treatment on fold change in mRNA expression in CD4+T cells treated with 5×10^−8^ M estradiol for (B) 2, (C) 6 and (D) 24 h. Control (no estradiol) is assigned a value of 1 (dashed line). UD = undetectable. The mean and SEM from 6 donors are shown.

Recognizing that the first cells to be protected by vaginal microbicides and most likely infected by HIV are CD4+T cells [48], we isolated purified blood CD4+T cells by magnetic bead separation prior to measuring nucleotidase gene expression. As shown in **Figure 7A** **,** in contrast to epithelial cells and fibroblasts, NT5C2 expression was greater than all other genes analyzed followed by NT5C3L and NT5C and NT5E. Interestingly, when relative expression of CD4+T cells was compared to that measured in epithelial cells and fibroblasts (not shown), all genes measured were 2–10 fold lower than that seen in cells from the FRT. Similar to our findings with fibroblasts, incubation of CD4+T cells with estradiol for 2, 6, or 24 h (**Figures 7B–D**), had no effect on nucleotidase gene expression.

#### Endothelial cells

Under conditions of oral ingestion of microbicides [48], TFV traverses and/or passes through endothelial cells to reach underlying immune cells in the FRT. To assess the extent to which estradiol regulates nucleotidase gene expression in endothelial cells, we analyzed two endothelial cell preparations. HUVEC cells were used in these studies in lieu of endothelial cells from FRT tissues, which are limited by the number of cells present in our tissue specimens. Irrespective of whether we analyzed primary HUVEC cells or the HUVEC cell line, we found that whereas epithelial cells and fibroblasts express 6/7 nucleotidase genes (**Figures 1** **and** **6**), HUVEC cells express 5/7 nucleotidase genes including NT5E, NT5C2, NT5C3L NT5C and NT5M. Interestingly, expression was significantly lower (approximately 10-fold) in HUVEC cells when compared to epithelial cells and fibroblasts (data not shown). To determine whether any of these genes were responsive to estradiol treatment, detailed time course studies (1–24 h) were carried out. Irrespective of the length of incubation and whether the primary or cell line were grown on inserts or plastic, estradiol had no effect on nucleotidase gene expression.

#### OAT1 and OAT3 transporters

In related studies, we determined that both sets of HUVEC cells as well as CD4+T cells, did not express the transporters OAT1 and OAT3 that would transport the microbicide into endothelial cells (data not shown), indicating that tenofovir enters tissues by other means than through endothelial cells in tissues. Similarly, neither the epithelial cells nor the fibroblasts from throughout the FRT expressed the genes for OAT1 and OAT3 (data not shown).

### Nucleotidase Biological Activity in FRT Epithelial Cells and Fibroblasts

**Figure 8:**
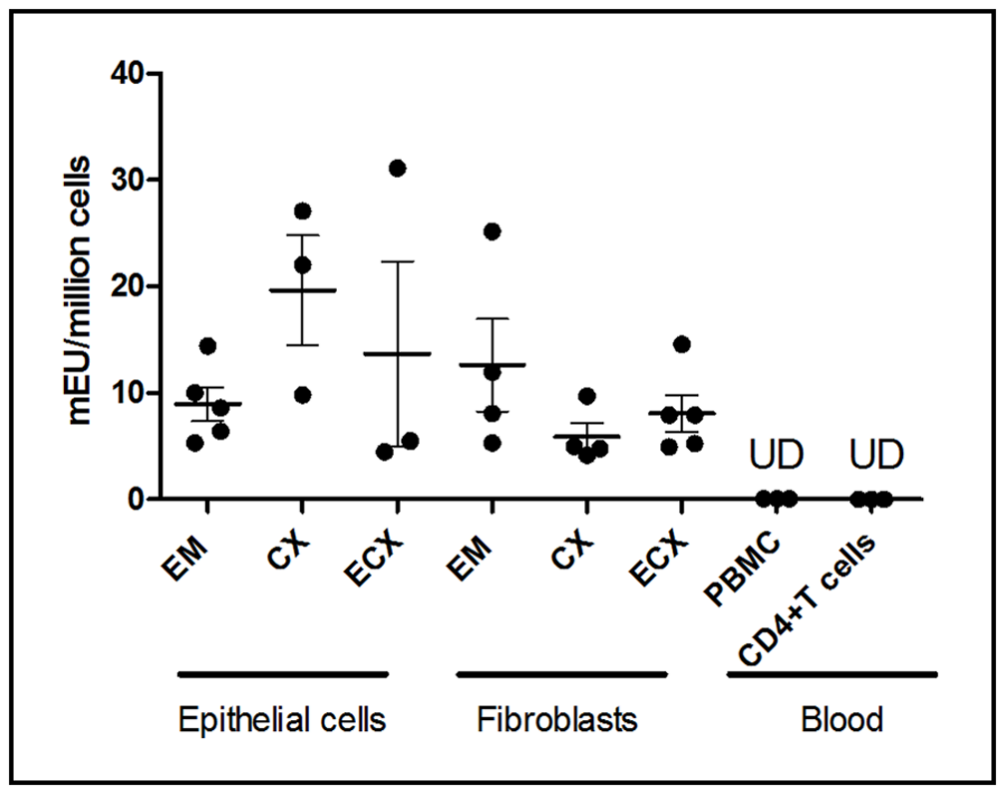
5′-Nucleotidase biological activity in epithelial cells and fibroblasts from the FRT, peripheral blood mononuclear cells (PBMC) and blood-derived CD4+T cells. 5′-nucleotidase biological activity was measured by a modified Diazyme 5′-nucleotidase assay in purified cultures of epithelial cells and fibroblasts from the EM, CX, and ECX as well as PBMC and CD4+T cells. Each circle represents tissue from an individual patient or donor. Values are expressed as milli Enzyme Units (mEU) per million cells. The mean and SEM are shown. UD = undetectable. Sensitivity of this biological activity assay is 0.05 mU per million cells.

5′-Nucleotidase, assayed as 5′-AMPase, has been extensively characterized and established as a stable, quantitative plasma membrane marker in blood cells as well as HeLa S3 cells [49]. To measure 5′-Nucleotidase biological activity in FRT cells, we adapted the Diazyme 5′-Nucleotidase assay from a serum to a cellular based assay to measure 5′-Nucleotidase biological activity in isolated epithelial cells and stromal fibroblasts from throughout the FRT. It is important to note that Nucleotidase biological activity, as measured by our modified assay, readily detects those nucleotidases that prefer purine-based substrates. These nucleotidases, which are thought to be involved in TFV-DP metabolism include NT5E, NT5C1A, and NT5C2. Nucleotidases with a preference for pyrimidine bases such as NT5C, NT5C3L, and NT5M would show very little activity with this assay. Following optimization of this assay in terms of cell numbers for each cell type, we evaluated nucleotidase activity in isolated cells and compared activity to that seen in PBMC and CD4+T cells isolated from blood. As seen in **Figure 8**, when 5′-Nucleotidase activity was measured, activity in epithelial cells and fibroblasts, but not PBMC or CD4+T cells, was measureable in cells from 3–5 FRT tissues. Interestingly, under conditions in which cell numbers were maximized to measure activity, biological activity was detected in purified CD4+T cells (data not shown). Moreover, we found that biological activity varies with the site analyzed. For examples, we found that activity tended to be greater in CX epithelial cells compared to that of EM or ECX epithelial cells. In contrast, the activity in fibroblasts was similar irrespective of whether cells were from the EM, CX and ECX.

### Effect of Estradiol on Nucleotidase Biological Activity in FRT Epithelial Cells and Fibroblasts

**Figure 9:**
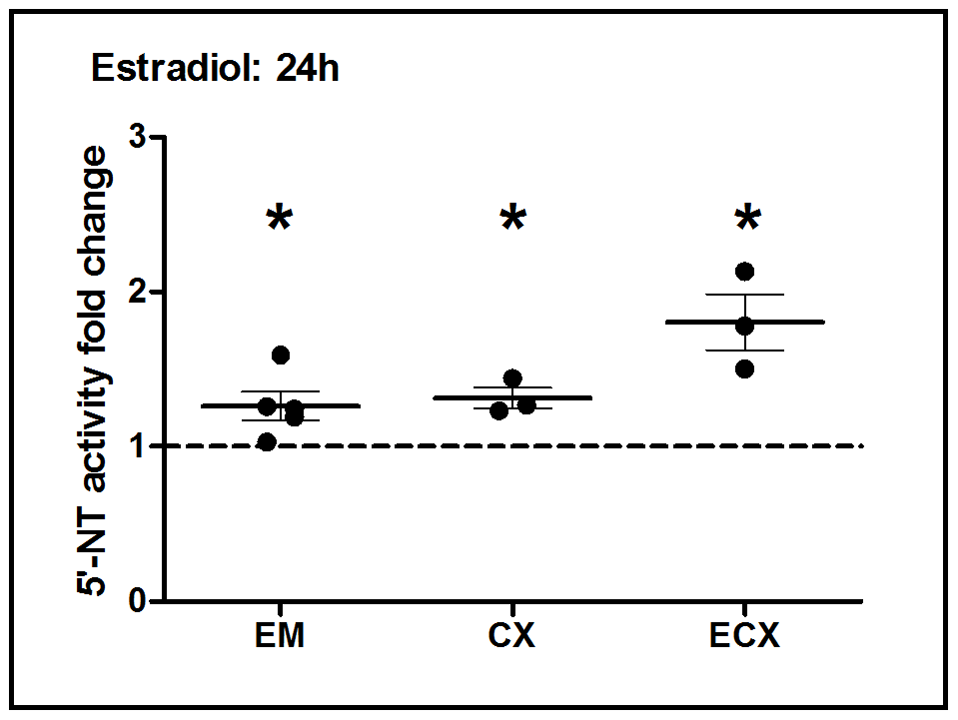
Effect of estradiol on 5′-nucleotidase biological activity in primary endometrial, endocervix and ectocervix epithelial cells. 5′-Nucleotidase biological activity was measured by a modified Diazyme 5′-Nucleotidase assay in purified cultures of EM, CX and ECX epithelial cells treated with estradiol (5×10^−8^ M) for 24 h. Data were normalized to control values, dashed line indicates an assigned value of 1. Each circle represents a different patient. The mean and SEM are shown. *, P<0.05 represents a significant difference between estradiol treatment and control.

To determine whether estradiol had an effect on 5′-Nucleotidase biological activity in primary FRT epithelial cells, cells were incubated with estradiol (5×10^−8^ M) for 24 h. As shown in **Figure 9**, when control values from 3–5 patients are normalized to 1.0, we found that E_2_ treatment of primary epithelial cells from the EM, CX and ECX significantly increased the 5′-nucleotidase biological activity by 30–70% at 24 h. To determine whether this effect persists in culture, EM cells were incubated with estradiol for 48 h prior to measurement of nucleotidase activity. We found that estradiol increases in expression at 48 h beyond that seen at 24 h (data not shown). Our findings of increased nucleotidase biological activity in epithelial cells are different from our mRNA gene expression findings for EM, CX and ECX epithelial cells. In contrast to EM epithelial cells, in which both biological activity and nucleotidase mRNA expression increased in response to estradiol (**Figures 2** **,** **3** **,** **4** **,** **5** **,** **9**), CX and ECX epithelial cell increases in biological activity occurred in response to estradiol despite the fact that we were unable to detect significant changes in mRNA nucleotidase expression. The apparent disconnect between gene expression fluctuations within 2–4 h and biological activity elevations at 24–48 hours may be due to cumulative expression of these genes, which results in an increase in biological activity.

**Figure 10:**
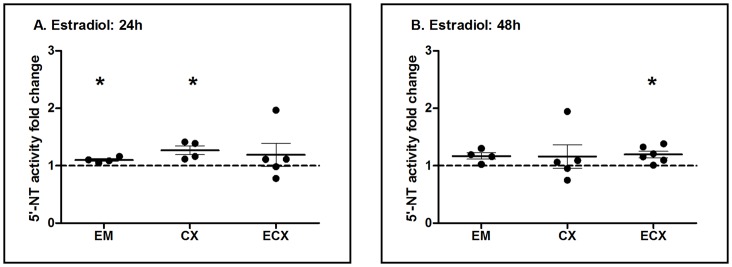
Effect of estradiol on 5′-nucleotidase biological activity in primary endometrial, endocervix and ectocervix fibroblasts. 5′-Nucleotidase biological activity was measured by a modified Diazyme 5′-Nucleotidase assay in purified fibroblasts. EM, CX and ECX fibroblasts were treated with estradiol (5×10^−8^ M) for (A) 24 and (B) 48 h. Data were normalized to control values, dashed line indicates an assigned value of 1. Each dot represents a different patient. The mean and SEM are shown. *, P<0.05 represents a significant difference between control and estradiol treatment at either 24 or 48 h.

In order to evaluate whether fibroblasts from the FRT are responsive to estradiol, isolated cells were incubated with estradiol for either 24 or 48 h prior to measurement of 5′-Nucleotidase biological activity. As shown in **Figure 10**, we found that fibroblasts from the EM, CX and ECX were hormonally responsive in that estradiol had a 24 h stimulatory effect on nucleotidase biological activity in EM and CX fibroblasts, as well as a 48 h increase in ECX fibroblasts. While responsiveness was more variable with time of incubation than that seen with epithelial cells, a stimulatory effect was seen in fibroblasts from each of 4–6 patients. This finding of increased biological activity was unexpected in light of our inability to measure changes in nucleotidase mRNA expression in response to hormone treatment.

Recognizing that progesterone is an important hormone present during the secretory phase of the menstrual cycle, we asked if progesterone alone and in combination with estradiol had any effect on 5′-Nucleotidase biological activity. In studies with epithelial cells and fibroblasts, we failed to observe any consistent response to progesterone alone or in combination with estradiol (data not shown). Similar findings were reported previously using histological staining of epithelial cells for nucleotidase enzyme [23].

### Nucleotidase Biological Activity in Blood CD4+T cells and Endothelial Cells

To determine whether CD4+T cell and HUVEC cell nucleotidase biological activity is altered by hormone treatment, cells were analyzed as described above following incubation with estradiol for 24 and 48 h. In several experiments in which the number of CD4+T cells was increased, biological activity was too low to measure with our assay. In contrast, when HUVEC cells were analyzed, nucleotidase activity was detectable and unaffected by estradiol treatment (not shown). Interestingly, HUVEC nucleotidase biological activity was approximately 5-fold less that that seen in epithelial cells and 2-fold less that measured in fibroblasts. Overall these results suggest that nucleotidase activity in endothelial cells is not hormonally regulated and unlikely to influence the metabolism of TFV in FRT tissues. Further studies are needed to determine whether estradiol regulates purified blood CD4+T cells metabolism of TFV-DP.

## Discussion

Tenofovir, either orally or applied locally to the vagina in gel form, has gained credence, albeit with some caveats, for the prevention of HIV infection in women [7], [48], [50]–[52]. To determine if sex hormones influence the expression and biological activity of enzymes that alter TFV metabolism, we evaluated the effect of estradiol on expression of NT gene expression and NT biological activity in purified human FRT epithelial cells and fibroblasts and found that FRT epithelial cells and fibroblasts from the FT, EM, CX and ECX express NT that are biologically active. Unlike epithelial cells from other FRT sites, estradiol increased NT gene expression in EM epithelial cells, but not epithelial cells or fibroblasts from other FRT sites. Using a biological assay for nucleotidases, estradiol increased NT activity of EM, CX and ECX epithelial cells and fibroblasts but had no effect on primary endothelial cells, an endothelial cell line, or blood CD4+T cells. Overall, these studies demonstrate that estradiol has a stimulatory effect on NT gene expression and/or biological activity in FRT epithelial cells and fibroblasts.

Nucleotidase biological activity as measured by our modified assay does not detect all of the different nucleotidase forms equally but readily detects nucleotidases such as NT5E, NT5C1A, and NT5C2 that prefer purine based substrates and would be expected to be involved in TFV-DP metabolism. Nucleotidases with a preference for pyrimidine bases such as NT5C, NT5C3L, and NT5M show very little activity with this assay. Our findings demonstrate that NT5E as well as 5 other nucleotidases are highly expressed in epithelial cells and fibroblasts, which make up the majority of cells in FRT tissues [53].

Our results demonstrate that estradiol increases both 5′NT nucleotidase biological activity and gene expression in EM epithelial cells. In contrast, estradiol increased 5′-NT biological activity in CX and ECX epithelial cells as well as EM, CX and ECX fibroblasts, but had no effect on 5′NT gene expression. One explanation for the apparent disconnect between gene expression and biological activity may be the cutoff for significance in the data presented. In previous studies, in which we measured FRT gene expression in FRT tissues from different individuals, we used a 2-fold change as a minimum for reporting significant differences [54]. In contrast, others have used less stringent conditions (1.5-fold), which magnifies differences seen in response to hormone treatment [55]. Using 1.5-fold, we found that a number of NT genes appear to increase (data not shown), but given the background expression and patient-to-patient variability we have reported these findings as not significant. Thus the cumulative expression of these genes might result in an increase in biological activity. What is clear from our uterine epithelial mRNA studies is that responsiveness to estradiol is rapid and transient (2 and 4 h). An alternative explanation is that despite our attempts to measure changes in nucleotidase activity, the time intervals selected may have been inadequate to detect transient changes in expression. Alternatively, since Christensen reported a lack of correlation between CD73 gene expression and ecto-5′nucleotidase bioactivity in blood mononuclear cells [56], her findings along with ours suggest that post-transcriptional mechanisms may be involved.

Our studies suggest that hormone regulation of endothelial cell nucleotidase mRNA and biological activity are unlikely to play a role in TFV metabolism to immune cells in the FRT. In addition to finding that nucleotidase gene expression in endothelial cells (primary and cell line) are lower (approximately 3-fold) than that seen in FRT epithelial cells and fibroblasts, under no conditions were we able to measure estradiol changes in nucleotidase mRNA expression or biological activity. Just why gene expression and biological activity were so low in our studies of endothelial cells is unclear. Endothelial cells are known to express NT5E that are stimulated by LPS [57] and inhibited by TNF-α [58]. It may be that under our culture conditions, endothelial cells are either not stimulated or that nucleotidase gene expression is negatively regulated and therefore express low levels of nucleotidases. Since endothelial cells are hormonally responsive [59], the failure of estradiol to modulate nucleotidase gene expression in the endothelial cells suggests that these genes may not be regulated in these cells. Alternatively, the lack of an estrogen effect on nucleotidases may be associated with the estrogen receptor profile since endothelial cells have ERβ and lack ERα [60]. Since these receptors have unique functions, it is possible that expression is not efficiently regulated through the ERβ system. Similar to endothelial cells, we found that NT mRNA expression in CD4+T cells was very low relative to FRT epithelial cells and fibroblasts and unresponsive to estradiol. Moreover, we found that we were unable to measure biological activity, under circumstances in which we know these cells are hormonally responsive to estradiol.

Given the importance of the menstrual cycle in gene expression, we asked whether estradiol, which peaks in blood at midway through the menstrual cycle [19], might influence the expression and biological activity of enzymes that alter TFV metabolism in the FRT. Others have shown that following entry by diffusion into cell, TFV is metabolized to TFV-DP, the active form that inhibits HIV reverse transcriptase [48], [51]. Our findings that estradiol increases NT biological activity in epithelial cells and fibroblasts suggest that TFV-DP could be converted back to TFV and diffuse or be transported out of these cells, thus making more TFV available for uptake by HIV-target cells. In the absence of estradiol, TFV-DP would be retained in epithelial cells and fibroblasts, which make up the majority of cells in the FRT. Under these conditions, we postulate that less TFV would be available for HIV-target cells protection. Whether epithelial cells and fibroblasts act as a reservoir for TFV that can provide microbicide to HIV-target cells in response to estradiol stimulation remains to be determined. Future studies of TFV metabolism are needed to determine whether changes of estradiol levels during the menstrual cycle influence microbicide protection against HIV infection during the menstrual cycle. Finally, whereas nucleotidase expression and bioactivity suggest a potential estradiol-induced effect on catabolism, metabolism of microbicides is an admixture of anabolic and catabolic events, both of which need to be addressed in future studies.

We previously hypothesized that women are most susceptible to HIV infection when estradiol levels are highest during the menstrual cycle [19]. This is due to estradiol suppression of specific immune mechanisms in the FRT designed to maximize the success of reproduction. Our finding that estradiol enhances NT biological activity in epithelial cells and fibroblasts suggests that HIV-target cells (CD4+T cells and macrophages) would be better protected at midcycle and during the early secretory stage of the menstrual cycle. These findings have major implications in that it would theoretically close the “Window of Vulnerability” in women using oral and topical PrEP. Of equal importance is the impact of chemical contraceptives on microbicide metabolism. In studies to determine TFV and TFV-DP concentrations in blood and blood PBMC, Coleman et al. found that the use of hormonal contraception (oral and injectable) was associated with decreased serum and intracellular PBMC TFV concentrations [61]. Since as many as 70% of women in TFV PrEP trials are using some form of chemical contraception, our findings that estradiol alters NT biological activity in cells from the FRT, when considered along with those of Coleman et al., provides compelling evidence that studies including microbicide trials are needed to determine the extent to which tissue in the FRT changes with stage of the menstrual cycle and hormonal contraceptive use.

Beyond its effects on TFV, our findings suggest that estradiol stimulation of NT activity in the human FRT may profoundly alter adenosine metabolism in FRT tissues. Adenosine is a significant signal molecule involved in a variety of physiological functions [62], many of which involve immune regulation [63]. For example, in neutrophils, ecto-5′-nucleotidase mediates the conversion of neutrophil-derived adenosine monophosphate to adenosine which promotes endothelial barrier function [64]. Also, in conjunction with CD39, which is an endothelial ecto-nucleoside triphosphate diphosphohydrolase (NTPDase), ecto-5′-nucleotidase produces extracellular adenosine, which affects neutrophil, macrophage and dendritic cell function [63]. Ecto-5′-nucleotidase is up-regulated by TGFβ in CD8+T cells, DCs and macrophages [65], and has been implicated in immunosuppression caused by T-regs [66], [67], macrophage activation [68], mucosal inflammation as well as Th17 immunosuppressive activity [69]. Other studies have shown that the CD39/adenosine axis is involved in T-reg suppression in HIV infection [67]. Additional studies are needed to examine the extent to which estradiol regulates adenosine modulation of immune protection against HIV infection.

In conclusion, these studies demonstrate that estradiol regulates NT expression and biological activity in epithelial cells and fibroblasts from the upper and lower FRT, but not in endothelial cells or blood CD4+T cells. Future studies are needed to evaluate the effects of sex hormones and chemical contraceptives on microbicide concentrations in FRT tissues as they relate to PrEP trial outcomes, to more fully define the complex interactions of the endocrine system and its influence on microbicide efficacy and protection against HIV.
